# MAb NJ001 inhibits lung adenocarcinoma invasiveness by directly regulating TIMP‐3 promoter activity via FOXP1 binding sites

**DOI:** 10.1111/1759-7714.13593

**Published:** 2020-08-03

**Authors:** Chunrong Gu, Ying Luo, Shichang Zhang, Jian Xu, Jiexin Zhang, Huanyu Ju, Jingping Liu, Lixia Zhang, Yan Zhang, Lei Wu, Erfu Xie, Ting Xu, Shiyang Pan

**Affiliations:** ^1^ Department of Laboratory Medicine The First Affiliated Hospital of Nanjing Medical University Nanjing China; ^2^ National Key Clinical Department of Laboratory Medicine Nanjing China

**Keywords:** Invasiveness, mAb NJ001, non‐small cell lung cancer, SP70 antigen, tissue inhibitor of metalloproteinase‐3

## Abstract

**Background:**

Previously, we developed a monoclonal antibody (mAb) NJ001 that binds to the antigen SP70 in human non‐small cell lung cancer (NSCLC) cells and showed it could inhibit lung adenocarcinoma (AD) growth. Here, we investigated the effect and mechanisms of NJ001 in lung AD metastasis.

**Methods:**

Human lung AD cells (SPC‐A1 and A549) were treated with different concentrations of mAb NJ001, and the effects of NJ001 on cell migration and invasive activity were investigated using wound‐healing and Matrigel assays, respectively. The molecular mechanism of this inhibition was explored by microarrays, qRT‐PCR, western blot, luciferase assays and electrophoretic mobility shift assays (EMSA).

**Results:**

MAb NJ001 markedly suppressed lung AD cell migration; and the invasiveness of SPC‐A1 and A549 cells treated with mAb NJ001 was diminished by 65%. Tissue inhibitor of matrix metalloproteinase‐3 (TIMP‐3) was highly expressed in SPC‐A1 cells treated with mAb NJ001, whereas knockdown of TIMP‐3 by shRNA significantly increased SPC‐A1 and A549 invasiveness. MAb NJ001 affects lung AD by inhibiting TIMP‐3 through direct transcriptional regulation of FOXP1 binding sites in the TIMP‐3 promoter region, as shown in luciferase assays and EMSA.

**Conclusions:**

MAb NJ001 inhibits invasiveness and metastasis in lung AD through the FOXP1 binding sites in the TIMP‐3 promoter region. It may have clinical applications in preventing and treating metastatic lung AD.

## Graphical abstract

The present study showed that mAb NJ001 inhibits lung AD cell migration and invasion in vitro, which were mediated by the FOXP1 binding sites in the TIMP‐3 promoter region. It may have clinical applications in preventing and treating metastatic lung AD.

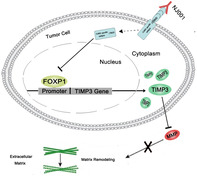

## Introduction

Lung cancer is the leading cause of cancer death worldwide and one of the 10 leading causes of death from all causes. Non‐small cell lung cancer (NSCLC) accounts for approximately 85% of all cases.^1^ Despite great progress in surgical techniques and chemoradiation therapy in recent years, NSCLC remains an aggressive malignant tumor with a five‐year survival rate of only ~15%.^2^, ^3^ The extremely poor prognosis of NSCLC is largely due to a high rate of distant metastasis after resection.

Monoclonal antibody (mAb) NJ001, which has high affinity and specificity for NSCLC cells, was generated in our laboratory.^4^, ^5^ Our previous studies showed that mAb NJ001 has effective antitumor activity both in vitro and in vivo.^5^ However, the effect of NJ001 on invasiveness and metastasis in lung adenocarcinoma (AD) has not yet been defined.

Tissue inhibitor of metalloproteinases (TIMPs) specifically inhibits the proteolytic activity of matrix metalloproteinases (MMPs), thus maintaining the balance of TIMPs and MMPs. TIMP‐3 is a possible tumor suppressor gene, which can tightly bind to the extracellular matrix and has been shown to inhibit tumor angiogenesis, invasion, and metastasis.^6^, ^7^ Clinical studies have shown that TIMP‐3 expression is decreased in many cancer types, and decreased expression of TIMP‐3 is significantly associated with pathologic stage, nodal involvement, and poor survival in lung cancer patients.^8^, ^9^

Here, we attempted to investigate the effects of mAb NJ001 on migration and invasion in lung AD cell lines, and the underlying molecular mechanisms were explored.

## Methods

### Cell lines and cell culture

Human lung fibroblast cell line HFL1 and human lung cancer cell lines SPC‐A1, A549, NCI‐H460 and NCI‐H520 were purchased from the cell bank of the Chinese Academy of Sciences in Shanghai. All cell lines were grown in RPMI 1640 medium supplemented with L‐glutamine, 100 units/mL penicillin, 100 mg/mL streptomycin and 10% (v/v) fetal bovine serum (FBS) (Gibco Invitrogen, Carlsbad, CA) at 37°C in a humidified 5% CO_2_ incubator. Cells were harvested for passaging or analysis using trypsin/EDTA.

### Microarray analysis

SPC‐A1 cells were seeded in six‐well plates at 2 × 10^6^ cells per well and, after overnight incubation, cultured with or without NJ001 (300 ng/μL) for 36 hours. Total RNA was extracted from SPC‐A1 cells using TRIzol (Invitrogen, Carlsbad, CA). Microarray analysis was performed using the GeneChip Human Genome 1.0 ST and the Two‐Cycle Target Labeling and Control Reagents kit (Affymetrix, Santa Clara, CA) according to the manufacturers' protocols. After hybridization of labeled mRNAs to mRNA microarrays, images were taken using an Affymetrix Gene Chip Scanner 3000. Data summarization, normalization and quality control were performed using the free mRNA QC Tool software. Comparison of samples at different times was based on the Wilcoxon rank‐sum test.

### Quantitative reverse transcription polymerase chain reaction (qRT‐PCR)

Total RNA was extracted and purified from cultured cells using the Qiagen miRNeasy Mini kit (Qiagen, CA, USA) according to the manufacturer's recommendations. Expression levels of MMP‐7 and TIMP‐3 were detected by quantitative reverse transcription polymerase chain reaction (qRT‐PCR). Primers for each gene were mmp‐7, forward 5′‐TCGAGACTTACCGCATATTAC‐3′, reverse 5′‐TCCAGCGTTCATCCTCAT‐3′; TIMP‐3, forward 5′‐CCTGCTGACAGGTCGCGTCT‐3′ reverse 5′‐TCCAGAGACACTCGTTCTTG‐3′. β‐actin was used as an internal control, with the primers 5′‐TGGCCCCAGCACAATGAA‐3′ and 5′‐CTAAGTCATAGTCCGCCTAGAAGCA‐3′. qPCR used the SYBR Green (TaKaRa Biotechnology Co. Ltd., Dalian, China) dye detection method on ABI StepOne Sequence Detection System under default conditions: 95°C for 10 seconds, and 40 cycles of 95°C for 5 seconds and 60°C for 34 seconds. The comparative Ct method was used to quantify the transcripts.

### Western blot

For western blot analyses, cells were lysed with RIPA lysis buffer (50 mM Tris‐HCl, 1% NP‐40, 0.25% Na‐deoxycholate, 50 mM NaCl, 1 mM EDTA, 1 mM PMSF, 1 mg/mL aprotinin, 1 mM Na_3_VO_4_, 1 mM NaF). Next, 25 mg of total protein was electrophoresed on a 12% SDS‐PAGE gel, transferred to a PVDF membrane (Amersham Pharmacia Life Science, USA), and blocked with 10% non‐fat milk in TBST. The PVDF membrane was incubated with anti‐TIMP‐3 (1:1000; Cell Signaling Technology, Inc., Beverly, MA, USA) and anti‐GAPDH antibody (Zhongshan Biological, Beijing, China) overnight at 4°C to confirm equivalent protein loading in each lane, followed by incubation with HRP‐conjugated goat‐anti‐rabbit IgG for one hour at room temperature, washed three times with TBST for 15 minutes each, and finally developed with ECL (Amersham Life Science) on X‐ray film.

### shRNA synthesis and vector construction

The TIMP‐3 siRNA and a negative control (with a scrambled sequence with no match to any known gene) were selected based on the full‐length cDNA of human TIMP‐3 mRNA using a siRNA design software by Ambion (Table 1). A siRNA against GAPDH was included as a positive control to verify transfection reliability, RNA extraction and gene expression quantification. shRNAs (shTIMP‐3, shNC and shPC) were synthesized and inserted into the pGPU6/GFP/Neo vector by Shanghai GenePharma Co., Ltd.

**Table 1:** The target sites and target sequences of shRNA plasmids

Plasmids target sequences	
	5′‐CACCGCCTTAAGCTGGAGGTCAACATTCAAGAGATGTTGACCTCCAGCTTAAGGCTTTTTTG‐3′
shTIMP‐3	5′‐GATCCAAAAAAGCCTTAAGCTGGAGGTCAACATCTCTTGAATGTTGACCTCCAGCTTAAGGC‐3′
shNC	5′‐CACCGTTCTCCGAACGTGTCACGTCAAGAGATTACGTGACACGTTCGGAGAATTTTTTG‐3′
	5′‐GATCCAAAAAATTCTCCGAACGTGTCACGTAATCTCTTGACGTGACACGTTCGGAGAAC‐3′
shPC	5′‐CACCGTATGACAACAGCCTCAAGTTCAAGAGACTTGAGGCTGTTGTCATACTTTTTTG‐3′
	5′‐GATCCAAAAAAGTATGACAACAGCCTCAAGTCTCTTGAACTTGAGGCTGTTGTCATAC‐3′

### Construction of luciferase reporter plasmids

In luciferase reporter plasmids, the human TIMP‐3 promoter sequences (from −702 to +18, relative to the transcription start site of the TIMP‐3 gene) were synthesized and constructed into the pGL3‐enhancer vector (Promega, Madison, WI, USA) by the Generay Company (Shanghai, China). All plasmids were confirmed by DNA sequencing. Luciferase reporter constructs containing wild‐type or mutant promoter sequences were named pTIMP‐3 (−702/+18), pTIMP‐3 (mutP53), pTIMP‐3 (mutFOXP1) and pTIMP‐3 (mutE2F) (Fig 4(a)).

### Transient transfection and luciferase assay

SPC‐A1 and A549 cells were transfected by Lipofectamine 2000 (Invitrogen, Carlsbad, CA, USA) with 4 μg of each constructed luciferase plasmid. Simultaneously, 10 ng pRL‐SV40 per well was also transfected as an internal control to correct the transfection efficiency. Beforehand, cells were seeded on 24‐well plates overnight to ensure 90%–95% confluence at the time of transfection. Then, 24 hours after transfection, transfected cells were treated with or without mAb NJ001 (100 ng/μL), and 48 hours after transfection, luciferase activity was measured by the Dual‐Luciferase Reporter Assay System (Promega, Madison, WI, USA) and expressed as the ratio of firefly luciferase to Renilla luciferase activity. All experiments were performed in triplicate.

### Electrophoretic mobility shift assay (EMSA)

Sense probe sequences were FOXP1 BS1 wild‐type probe, 5′‐AGAACTCTGTTTTGTTTTCTTCAT‐3′; FOXP1 BS1 mutant probe, 5′‐GAGCACAGAAAACAGTCTTCTATC‐3′; FOXP1 BS2 wild‐type probe, 5′‐AGAACTCCCGGTCCGGTTCTTCAT‐3′; FOXP1 BS2 mutant probe, 5′‐GAGCACAGAAGGGCGTCTTCTATC‐3′. Nuclear proteins were extracted with NE‐PERTM Nuclear and Cytoplasmic Extraction Reagents (Pierce, Rock‐ford, IL, USA). DNA probes were prepared with the Biotin 3′‐End DNA Labeling Kit (Pierce). EMSA was performed with a LightShift Chemiluminescent EMSA Kit (Pierce). Binding reactions were performed with nuclear extracts (8 mg) and binding buffer with 2.5% glycerol, 5 mM MgCl_2_, 50 ng/mL poly (dI‐dC), 0.05% NP‐40, and 20 fmol biotin‐labeled FOXP1 BS1/FOXP1 BS2 probes, which were incubated on ice for 30 minutes in a volume of 20 mL. For competition studies, nuclear extracts were incubated with unlabeled oligonucleotides for 30 minutes before adding the labeled oligonucleotides.

### Tumor cells invasion assay

SPC‐A1 and A549 cells were transfected with shTIMP‐3 or treated with mAb NJ001 (0, 50, 100 and 150 ng/μL) for 24 hours, then harvested and placed into transwell inserts that contained 500 μL of RPMI 1640 media plus 10% FBS in the lower chamber. After 24 hours, cells that had passed through the Matrigel membrane were stained with crystal violet and counted (8 high‐power fields, × 200 magnification). Experiments were conducted in triplicate.

### Wound‐healing assay

SPC‐A1 cells in six‐well plates were cultured until monolayers formed. After being starved overnight, SPC‐A1 cells were exposed to mitomycin C (10 μg/mL) for two hours. Cell layers were wounded using a 10 μL pipette tip and cultured with mAb NJ001 (0, 50, 100 or 150 ng/μL) for another 48 hours. Photographs were taken at 0 and at 48 hours. The distance traveled by the cells was measured between the two boundaries of the acellular area and the results of the treatment groups were expressed as a ratio to the control group cells. Each experiment was performed in triplicate.

### Immunoelectron microscope

SPC‐A1 and HFL1 cells were cultured in six well plates. The cells were fixed and stained over night with the mouse mAb NJ001 (1:400) in 2% normal goat serum, washed with 50 mM TBS and stained for 30 minutes with a gold‐coupled antimouse antibody (1:50, Nanoprobes, Stony Brook, New York, USA). Samples were analyzed with a Zeiss Leo TEM. Briefly, pictures of cells were randomly taken, the regions of interest were outlined and the number of gold particles was counted within these regions. Nontreated, stained cells of the same culture dishes were taken as negative control.

### Cytoplasmic, nuclear and membrane protein extraction

Treated SPC‐A1 cells (treated with 200 ng/μL NJ001 for 48 hours) and control SPC‐A1 cells were collected to extract cytoplasmic, nuclear and membrane protein using Compartmental Protein Extraction Kit (Millipore, Boston, Massachusetts, USA) according to the manufacturer's recommendations. Cold buffer C was added to SPC‐A1 cells and rotated at 4°C for 20 minutes. Following centrifugation (15 000 *g* for 20 minutes at 4°C), supernatants were kept as cytoplasmic proteins and pellets were washed once with buffer W. Cell pellets were then incubated in Buffer N and rotated at 4°C for 20 minutes. Following centrifugation (15 000 *g* for 20 minutes at 4°C), supernatants were kept as nuclear proteins and pellets were incubated in Buffer M to isolate membrane proteins.

### Co‐immunoprecipitation (Co‐IP)

Co‐IP was carried out using the Pierce Co‐Immunoprecipitation Kit (Pierce, Rock‐ford, Illinois, USA) according to the manufacturer's recommendations. A total of 1 mg of nuclear protein of SPC‐A1 treated with or without mAb NJ001 was incubated with equal amounts (3 μg) of FOXP1 antibody or normal rabbit IgG (Cell Signaling Technology Inc., Beverly, Massachusetts, USA) and protein A‐Agarose preblocked with BSA. FOXP1 immunocomplexes eluted by nonreducing SDS buffer were resolved on 10% polyacrylamide gels and immunoblotted with mAb NJ001 (1:1000).

### Statistical analysis

The results are expressed as mean ± s.d. Differences between experimental groups were analyzed with the χ^2^ test. For single comparisons of two groups, Student's *t*‐test was used. *P* < 0.05 was considered statistically significant.

## Results

### MAb NJ001 inhibits migration and invasiveness of SPC‐A1 cells

**Figure 1:**
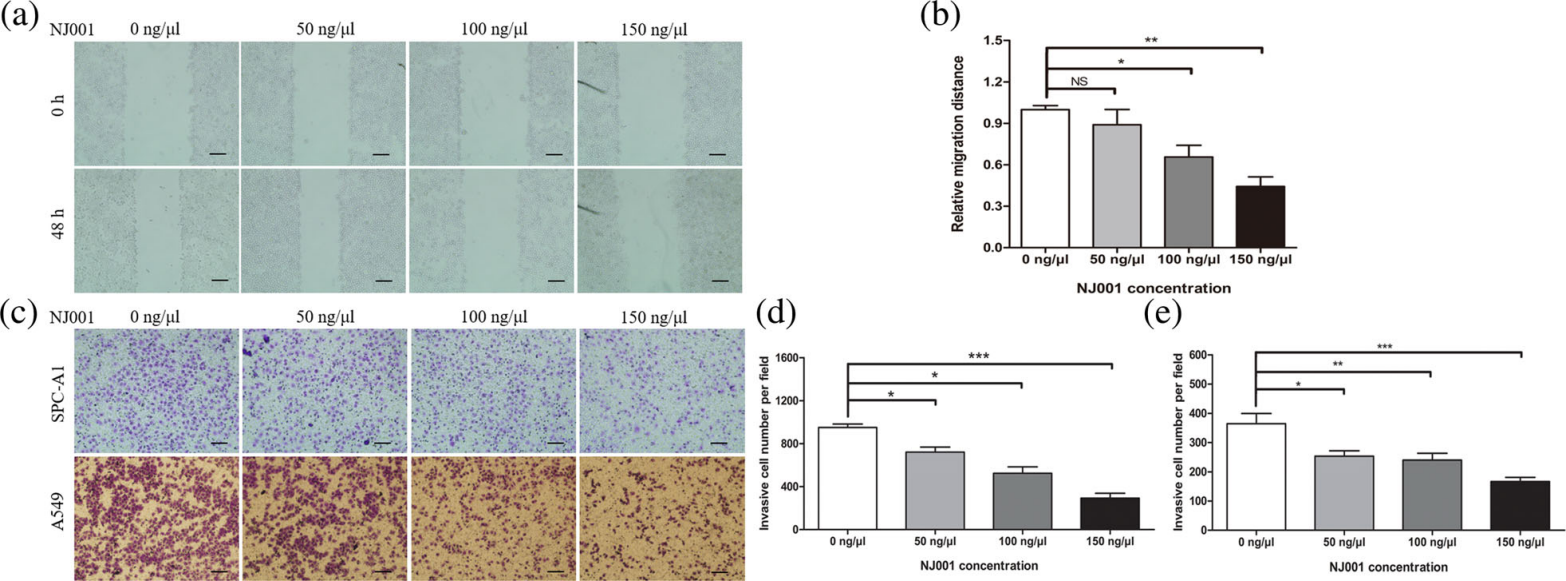
Effect of mAb NJ001 on lung adenocarcinoma cell motility and invasiveness. (**a**) Representative images of SPC‐A1 cell motility under treatment with 0, 50, 100 or 150 ng/μL NJ001 at 0 and 48 hours, respectively. Scale bar: 100 μm. (**b**) Cell motility was quantified by measuring the distance traveled by the cells between the two boundaries of the acellular area; the results are expressed as a ratio to cells treated with 0 ng/μL NJ001 (controls). (**c**) Representative images of SPC‐A1 and A549 cell invasiveness and metastatic ability after treatment with 0, 50, 100 or 150 ng/μL NJ001 for 48 hours. Scale bar: 100 μm. (**d, e**) Cell invasiveness of (**d**) SPC‐A1; and (**e**) A549 cells was quantified by counting cells that passed through the Matrigel membrane, using a light microscope (×200). Each experiment was performed three times. Data are expressed as the means ± s.d. *P < 0.05, **P < 0.01, compared with 0 ng/μL NJ001.

SPC‐A1 cells were treated with mAb NJ001 for 48 hours. NJ001 slowed cell migration across the wound scratch in a dose dependent manner (Fig 1(a)). Quantitative analyses at 48 hours confirmed a significant reduction in wound closure in cells treated with 100 and 150 ng/μL NJ001 compared with the control cells (0 ng/μL NJ001) (Fig 1(b)).

To study the effect of mAb NJ001 on invasiveness in lung AD cells, we used a Matrigel model (Fig 1(c)). Movement by SPC‐A1 and A549 cells treated with 150 ng/μL NJ001 was significantly impaired, by up to 65% at 24 hours (Fig. 1(d,e)).

### MAb NJ001 increased expression of metastasis‐related gene tissue inhibitor of metalloproteinases 3 (TIMP‐3) in SPC‐A1 cells

To elucidate the mechanisms underlying the inhibitory effect of mAb NJ001 on lung AD cell invasiveness, we used an Affymetrix Human 1.0ST gene chip assay to analyze gene expression (GSE59655, https://www.ncbi.nlm.nih.gov/geo/). Compared with control cells, mAb NJ001 altered downstream target genes matrix metallopeptidases 7 (MMP‐7) and tissue inhibitor of metalloproteinases 3 (TIMP‐3), both of which regulate cancer invasiveness and metastasis. MAb NJ001 increased the expression of TIMP‐3 (2.0‐fold) and suppressed MMP‐7 (−2.1‐fold) (Table 2).

**Table 2:** Effect of mAb NJ001 on gene expression in SPC‐A1 cells

Full name	Gene	GeneBank	Fold change (NJ001/control)	Function
Metallopeptidase inhibitor 3	TIMP‐3	NM_000362	2.0	Extracellular matrix protein
Matrix metallopeptidase 7	MMP‐7	NM_002423	−2.1	Extracellular matrix protein

**Figure 2:**
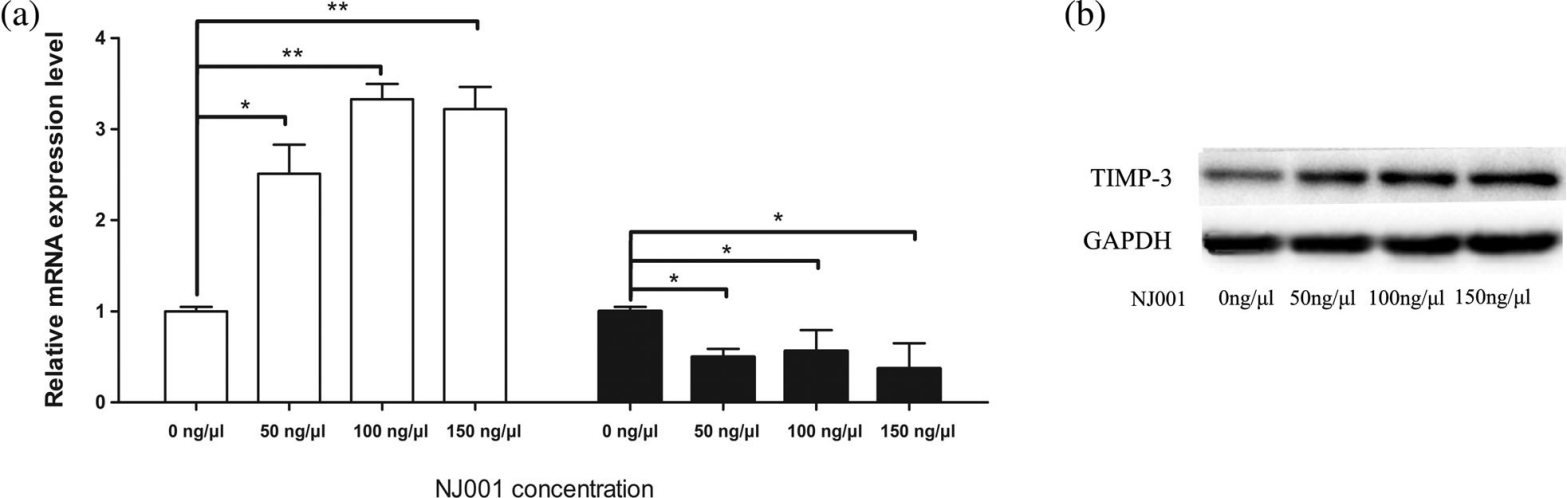
Effect of mAb NJ001 on downstream gene expression. (**a**) RT‐qPCR analyses were used to validate TIMP‐3 and MMP‐7 expression. Data are expressed as the means ± s.d. *P < 0.001, compared with 0 ng/μl NJ001. (**b**) Western blots confirmed TIMP‐3 expression regulated by mAb NJ001 in SPC‐A1 cells. GAPDH was used as an internal control. Lower panel: relevant band densitometry analysis.

To validate these gene chip results, quantitative PCR and western blot were performed on SPC‐A1 cells treated with mAb NJ001 (at 0, 50, 100 or 150 ng/μL). MAb NJ001 significantly increased TIMP‐3 and suppressed MMP‐7 mRNA expression in SPC‐A1 cells (Fig 2(a)). TIMP‐3 protein expression was also enhanced by mAb NJ001 (Fig 2(b)), but MMP‐7 protein could not be detected in SPC‐A1 cells. Thus, TIMP‐3 may play a larger part than MMP‐7 did in inhibiting the invasiveness of mAb NJ001‐treated lung AD cells.

### TIMP‐3 mediates inhibition of invasiveness in mAb NJ001‐treated lung AD

**Figure 3:**
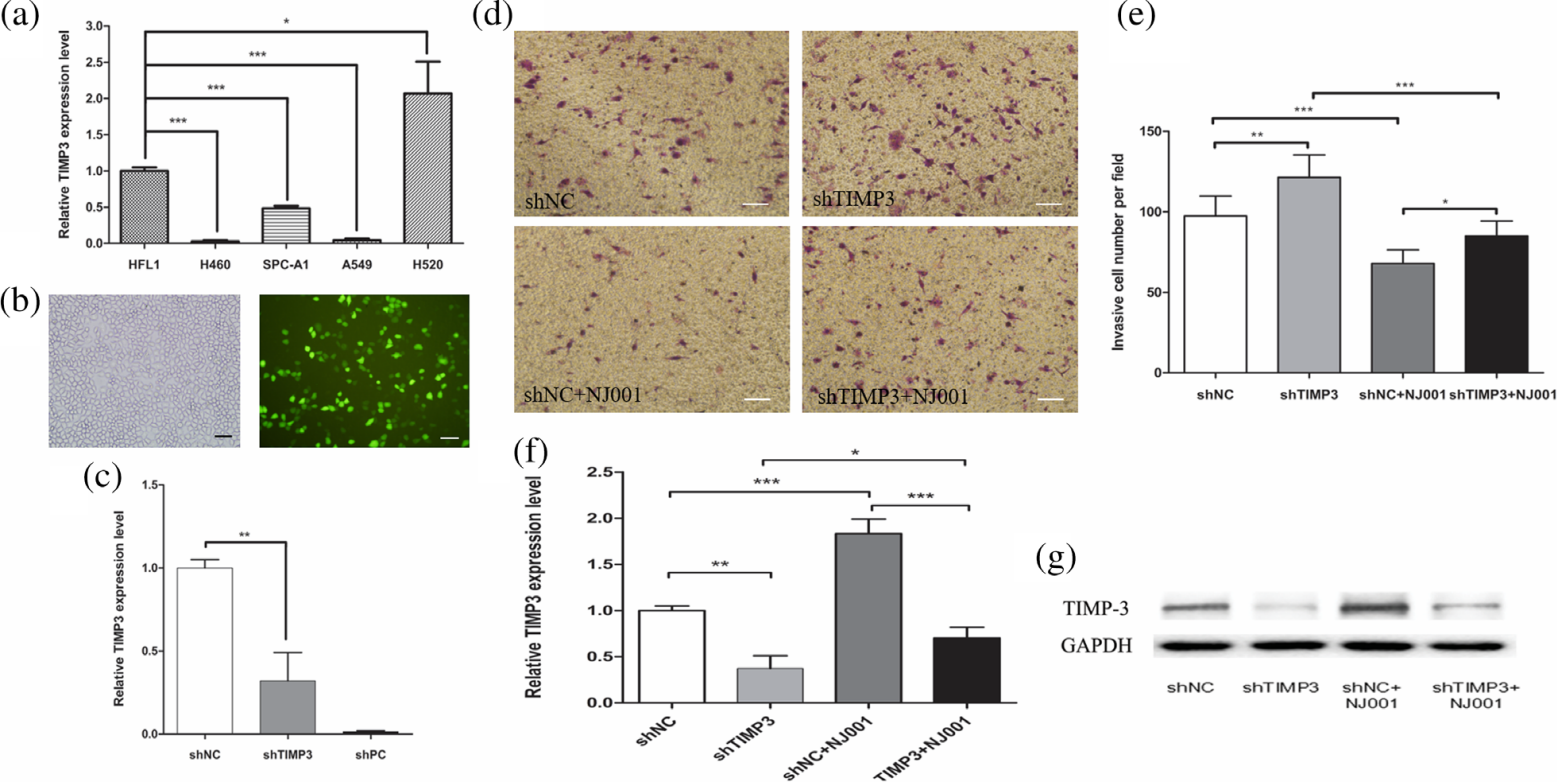
Effect of TIMP‐3 on invasiveness of lung adenocarcinoma cells treated with mAb NJ001. (**a**) Detection of endogenous TIMP‐3 in human lung cancer cell lines (SPC‐A1, A549, H460 and H520) and human lung fibroblast cell line HFL1, using RT‐qPCR. Data are expressed as the means ± s.d.
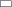
TIMP‐3, 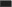
MMP‐7; *P < 0.001, compared with HFL1 cells. (**b**) SPC‐A1 cells were transfected with shTIMP‐3 plasmid containing a GFP gene and viewed under a fluorescence microscope. The left is phase‐contrast microscopic image of transfected SPC‐A1 cells, and the right is fluorescence microscopic image of transfected SPC‐A1 cells at the same field. Scale bar: 100 μm. (**c**) RT‐qPCR was used to analyze the TIMP‐3 mRNA level in SPC‐A1 after transfection with shTIMP‐3 for 48 hours. Data are expressed as the means ± s.d.; *P < 0.001, compared with shNC. (**d**) Representative images of invasiveness and metastasis in SPC‐A1 cells transfected with shTIMP‐3 and/or treated with 100 ng/μL NJ001 at 48 hours. Scale bar: 100 μm. (**e**) Cell invasion was quantified by counting cells that passed through the Matrigel membrane, using a light microscope. Experiments were performed three times. Data are expressed as the means ± s.d.; *P < 0.05, **P < 0.01, compared with shNC. (**f**) The TIMP‐3 mRNA level in SPC‐A1 was analyzed by RT‐qPCR after transfection with shTIMP‐3 and/or treatment with 100 ng/μL NJ001. (**g**) Western blots confirmed TIMP‐3 expression after transfection with shTIMP‐3 and/or treatment with 100 ng/μL NJ001 in SPC‐A1. GAPDH was used as an internal control. Lower panel: relevant band densitometry analysis.

To further determine the role of TIMP‐3 in inhibiting invasiveness of mAb NJ001‐treated lung AD cells, we developed SPC‐A1 cells with endogenous TIMP‐3 knockdown, using shTIMP‐3. We selected SPC‐A1 cells for the TIMP‐3 silencing and tumor cell invasion assay because their endogenous TIMP‐3 level is lower than that of human lung fibroblast cells (HFL1) (Fig 3(a)), which is similar to TIMP‐3 expression in tumor cells from patients with lung cancer.^9^ At 48 hours after transfection with shRNA, expression of pGPU6/GFP/Neo which included a cassette of green fluorescent protein showed >60% transfection efficiency (Fig 3(b)), and expression of TIMP‐3 mRNA was decreased by 70% (Fig 3(c)). In mAb NJ001‐treated SPC‐A1 cells, TIMP‐3 knockdown successfully reversed the inhibitory effect of mAb NJ001 on cancer cell invasiveness (Fig 3(d,e)). The combination of mAb NJ001 and shTIMP‐3 reduced the expression of both TIMP‐3 mRNA and protein (Fig 3(f,g)).

### Transcriptional regulation of TIMP‐3 by mAb NJ001

**Figure 4:**
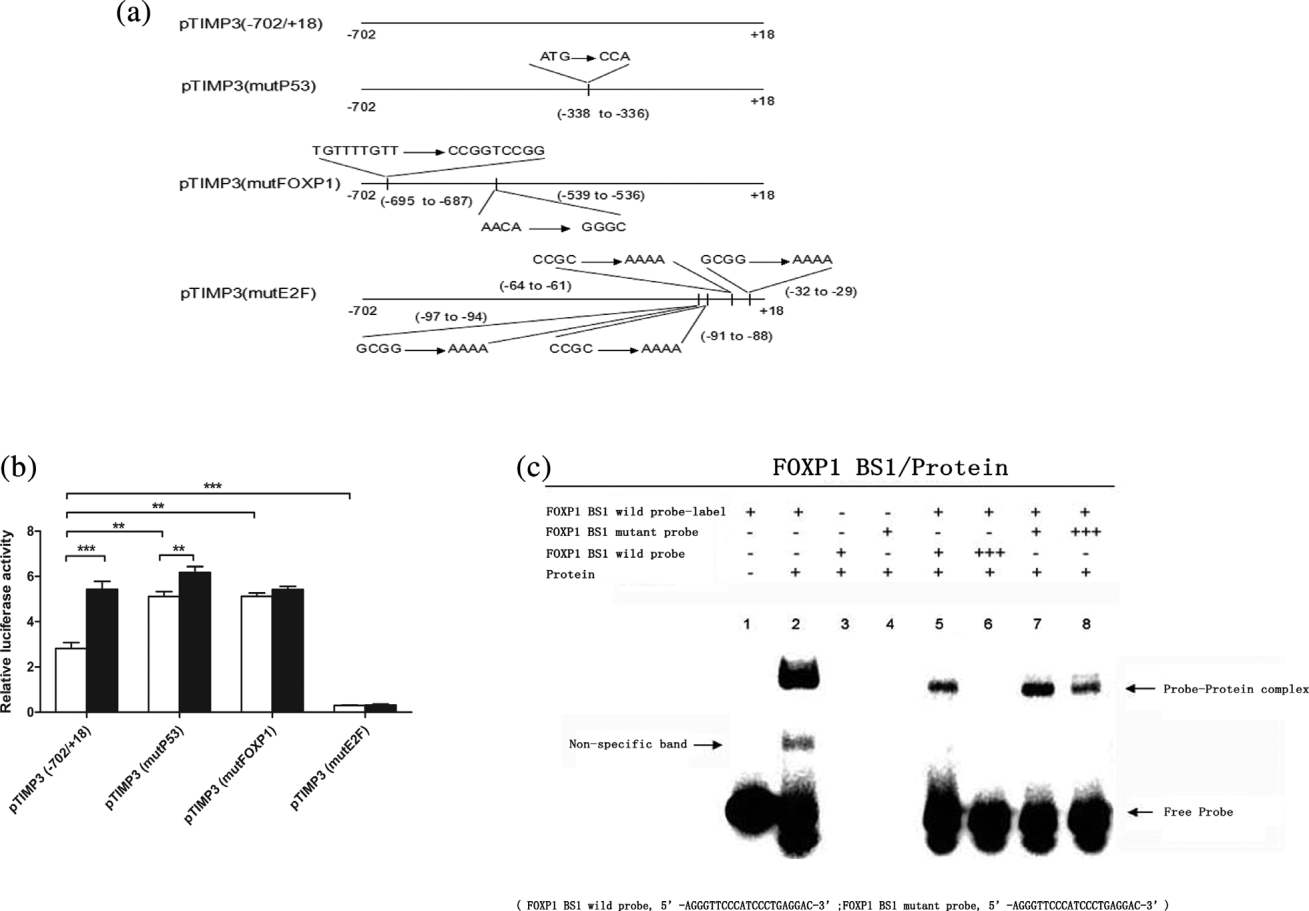
Transcriptional regulation of mAb NJ001 on the TIMP‐3 gene in SPC‐A1 cells. (**a**) The DNA sequences of TIMP‐3 promoter region were cloned into luciferase reporter constructs: pTIMP‐3 (^−^702/+18), pTIMP‐3 (mutP53), pTIMP‐3 (mutFOXP1) and pTIMP‐3 (mutE2F). (**b**) TIMP‐3 promoter activity was analyzed with a luciferase reporter assay in SPC‐A1 cells. Data are expressed as the means ± s.d. 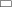
Control, 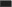
NJ001; *P < 0.001, compared with pTIMP‐3 (^−^702/+18) or luciferase reporter constructs not treated with NJ001. (**c**) Electrophoretic mobility shift assay (EMSA) to analyze the activity of nuclear proteins at FOXP1 binding sites in the TIMP‐3 promoter region. Biotinylated probes (20 fmol) were incubated with 8 mg of nuclear extracts from SPC‐A1 cells. In competition experiments, 10‐, 100‐, and 200‐fold molar excess of unlabeled A/B probes show the specificity of each binding reaction.

To further determine whether the mAb NJ001‐mediated change in TIMP‐3 expression occurs on the transcriptional level, the TIMP‐3 promoter region was analyzed using MatInspector software (Genomatrix Software GmbH, Munich, Germany). Four luciferase reporter plasmids containing wild‐type TIMP‐3 promoter sequences or P53/FOXP1/E2F binding site mutant sequences were synthesized (Fig 4(a)). Promoter binding sites for P53 and FOXP1 transcription factors are negative regulators of TIMP‐3, whereas binding sites for E2F strongly enhance TIMP‐3 activity. MAb NJ001 directly activates TIMP‐3 transcription by suppressing the FOXP1 binding sites (Fig 4(b)). We performed EMSA in SPC‐A1 cells (Fig 4(c)) and observed a strong protein–probe band to shift when the nuclear extracts were incubated with TIMP‐3 BS1 or TIMP‐3 BS2 wild biotin‐labeled probes. The protein–probe band could not compete with mutant probes (Fig 4(c)). These findings suggest that mAb NJ001 directly regulates TIMP‐3 expression on the transcription level.

### Mab NJ001 increased SP antigen binding to FOXP1 in cell nucleus

**Figure 5:**
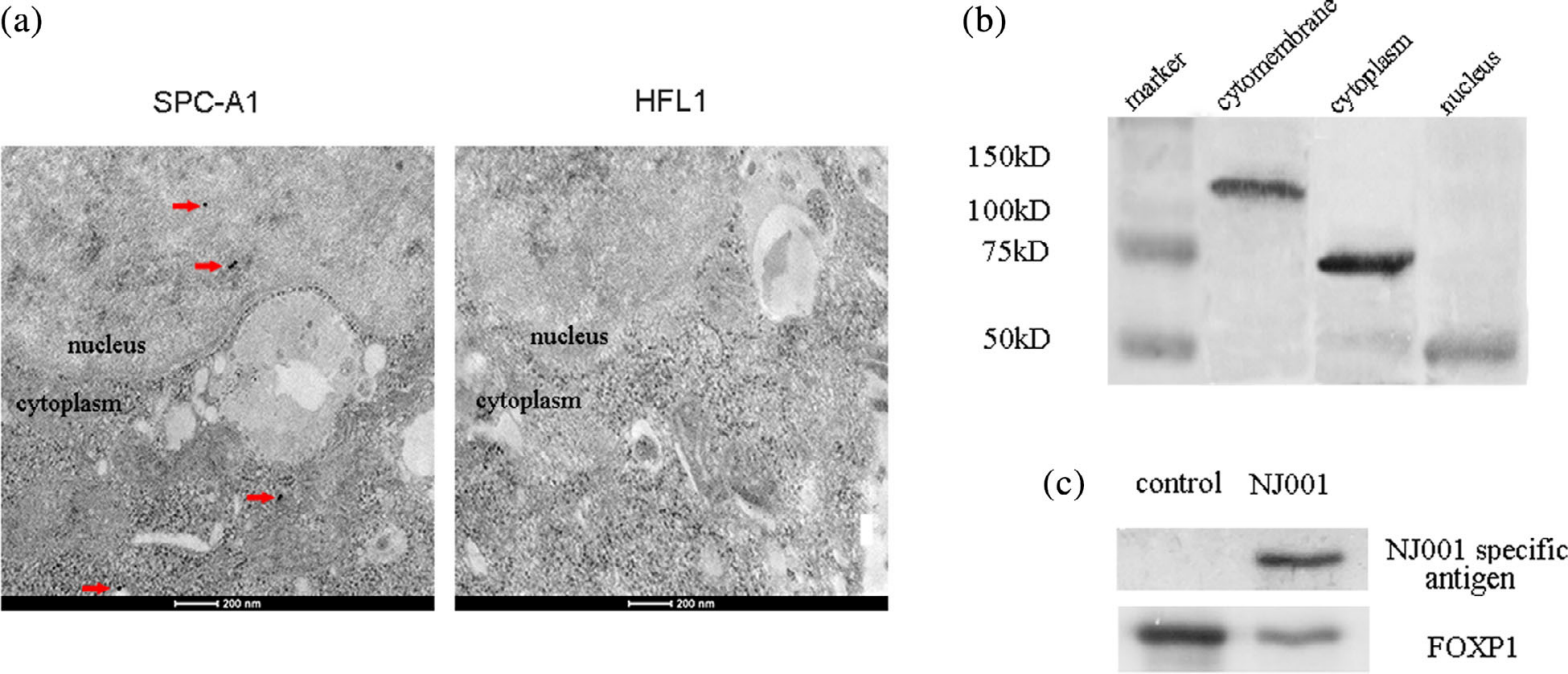
Effect of mAb NJ001 on its specific antigen in SPC‐A1 cells. (**a**) Subcellular localization of NJ001 specific antigen was analyzed using immunoelectron microscope. Dots represent NJ001 specific antigens. (**b**) Western blots confirmed NJ001 specific antigen expression in cytoplasm, cell nucleus and cytomembrane of SPC‐A1. (**c**) The nuclear protein of control SPC‐A1 and SPC‐A1 treated with NJ001 were immunoprecipitated with an anti‐FOXP1 antibody or anti‐rabbit IgG, as control. The expression of NJ001 specific antigen in the immune complexes was determined by western blot using mAb NJ001.

To identify the subcellular location of NJ001 specific antigens, immunoelectron microscope was applied, and it showed that gold particles of NJ001 specific antigens were in cytoplasm and cell nucleus of SPC‐A1 (Fig 5(a)). However, none of the gold particles were found in HFL1 cells (Fig 5(a)). Western blot confirmed that NJ001 specific antigens were located in the cytomembrane, cytoplasm, and cell nucleus, and the molecular weights of SP antigens were 120, 70 and 50 kDa respectively (Fig 5(b)). As FOXP1 is a transcriptional regulator, the interaction between NJ001 specific antigen and FOXP1 is unclear. Co‐immunoprecipitation and western blot showed that the NJ001 specific antigens (about 50 kDa) were expressed in FOXP1 immune complexes in nucleus of NJ001 treated SPC‐A1 cells (Fig 5(c)). These findings suggest that mAb NJ001 induces its specific antigens and FOXP1 to form the immune complexes in nucleus of LAD cells.

## Discussion

NJ001 is a newly‐developed monoclonal antibody that is specific to NSCLC. Our previous studies showed that mAb NJ001 has effective antitumor activity.^5^ However, the effect of mAb NJ001 on invasiveness and metastasis in lung AD is not well studied. Here, we showed that mAb NJ001 inhibited metastatic activity in lung AD cell lines (SPC‐A1 and A549), particularly in wound healing, cell migration and invasion. However, the molecular mechanisms of these effects in lung AD cells have not been previously explored. To elucidate the main regulators of NJ001‐mediated inhibition of metastasis in lung AD, we used a cDNA microarray to study mAb NJ001‐treated SPC‐A1 cells; genes with significantly altered expression levels were then validated by qRT‐PCR and immunoblotting. We found that the inhibition of invasiveness and migration in mAb NJ001‐treated cells was mediated by upregulation of TIMP‐3.

TIMP‐3 is an important endogenous inhibitor of matrix metalloproteinases, and of angiogenesis.^10^, ^11^ Decreased levels of TIMP‐3 have been shown in various human malignancies including breast cancer, prostate cancer, gastric cancer, esophageal squamous cell carcinoma, endometrial cancer, and cholangiocarcinomas.^12^, ^13^, ^14^, ^15^, ^16^ Low TIMP‐3 expression has been associated with a poor prognosis and nodal metastasis in various cancers.^8^, ^17^, ^18^, ^19^ Unrelated to its inhibitory effect on metalloproteinases, TIMP‐3 is thought to inhibit vascular epithelial growth factor (VEGF)‐mediated angiogenesis by blocking the binding of VEGF to its receptor.^10^, ^20^ TIMP‐3 overexpression induces apoptosis in stromal cells.^21^, ^22^, ^23^ TIMP‐3 also affects extracellular matrix signaling.^24^

In the present study, upregulation of TIMP‐3 expression in mAb NJ001‐treated cells suggests it has a role in suppressing lung AD invasion and metastasis. We have shown that endogenous TIMP‐3 knockdown reverses the inhibitory effect of mAb NJ001 on cancer cell invasiveness, which indicates thatTIMP‐3 is a key regulator of the antimetastatic function of mAb NJ001. To further investigate the regulatory mechanism of TIMP‐3 by mAb NJ001, we investigated three transcription factor binding sites on the TIMP‐3 gene promoter in SPC‐A1 cells and found that P53 and FOXP1 negatively regulates TIMP‐3 promoter activity, whereas E2F positively regulates TIMP‐3 promoter activity. We have also shown that mAb NJ001 enhances the transcription of TIMP‐3 by directly regulating TIMP‐3 promoter activity via FOXP1 binding sites.

Forkhead Box P1 (FOXP1) is a widely expressed transcription factor that reportedly plays an important role in several types of solid tumors.^25^, ^26^, ^27^, ^28^, ^29^, ^30^ FOXP1 is considered to be an oncogene in some malignancies.^31^, ^32^, ^33^, ^34^, ^35^ Here, we found that FOXP1 inhibits TIMP‐3 promoter activity in lung AD. It is a molecular target of mAb NJ001 in the regulation of TIMP‐3 expression, further inhibiting tumor invasion.

In conclusion, mAb NJ001 suppresses lung AD cell migration and invasion in vitro, and these effects are mediated by TIMP‐3. These results imply that mAb NJ001 could have clinical applications in preventing or treating metastatic lung adenocarcinoma.

## Disclosure

The authors declare no conflicts of interest.
